# *Ascosphaera callicarpa*, a New Species of Bee-Loving Fungus, with a Key to the Genus for Europe

**DOI:** 10.1371/journal.pone.0073419

**Published:** 2013-09-25

**Authors:** Anja A. Wynns, Annette B. Jensen, Jørgen Eilenberg

**Affiliations:** Center for Social Evolution, Department of Plant and Environmental Sciences, University of Copenhagen, Frederiksberg, Denmark; California Department of Public Health, United States of America

## Abstract

We studied the bee specialist fungus *Ascosphaera* in wild solitary bees to investigate the diversity of the genus in nature and the ecology of these fungi with their bee hosts. A new morphologically distinctive species was discovered which also has a unique nrITS sequence. This new species, here named *Ascosphaera callicarpa*, is common on the larval feces of the solitary bee *Chelostoma florisomne* which nests in the *Phragmites* reeds of thatched roofs in Europe. Because collections of *Ascosphaera* from wild bees are scarce and because little is known about the ecology and distribution of the majority of the species in the genus, a key to the species thus far reported for Europe is included.

## Introduction

*Ascosphaera* is a genus of 28 species of bee specialist fungi with a worldwide distribution in the temperate to tropical regions. The genus is remarkable for its host and habitat specificity with all species completing their entire life cycle within the nests of bees (Apoidea: Anthophila). *Ascosphaera* was first discovered in the early 20^th^ century in Europe after *A. apis*, the type species, was identified as the causative agent of a brood disease affecting honeybees [1], [2]. This brood disease, known as chalkbrood, was later observed in a solitary bee in London [3]. *Ascosphaera* is widely known as the chalkbrood fungus, although at least half of the species lead a saprotrophic rather than pathogenic lifestyle [4], [5]. Saprotrophic *Ascosphaera* species flourish on diverse substrates within the bee nest, for example on pollen provisions, on materials used by the bees to construct the nest and on larval feces [6], [7]. Little is known about these saprotrophs which appear to live innocuously inside the brood cells of the bees. Consequently, the potential for research on the ecological and functional role of these fungi within the bee nest remains wide open.

*Ascosphaera* is placed in Ascosphaeraceae (Pezizomycotina: Eurotiomycetidae), a small family of ascomycetes primarily characterized by a unique fruiting body type called a spore cyst. Spore cysts are unicellular, cyst-like fruiting bodies that form from the expansion of a single cell called a nutriocyte [8]. The wall of a spore cyst is a double-layered membrane. Asci are free-floating and evanescent. Because of their anomalous fruiting bodies, the taxonomic affinities of *Ascosphaera* and its relatives remained uncertain until ontological studies led C.F. Spiltoir and L.S. Olive [8] to confidently place them among the Ascomycota within Eurotiomycetidae [as Plectascales]. This position was later confirmed by additional morphological study [9] and DNA sequenced-based phylogenies [10], [11].

A distinguishing feature of *Ascosphaera* is the presence of spore balls [8]. A spore ball is a compact aggregation of spores formed by groups of asci that are united by a single membrane [12]. The membrane surrounding a spore ball disintegrates and only remnants of it are sometimes observed in mature spore cysts [5]. Spore balls may contain as few as two to as many as several hundred ascospores [5], [13]. The average number of ascospores per spore ball and the persistence of spore balls at maturity are meaningful taxonomic characters.

Pathogenic *Ascosphaera* species afflict only the larval stage of bees. Typically diseased larvae die in the larval stage; however, in rare occurrences, larvae have been observed to enter pupation before being overcome by the fungus (Wynns pers. obs.). Pathogenic species of *Ascosphaera* appear to be highly specialized fungi with ascospores typically germinating only when within the midgut of their host. Spore germination is followed by rapid hyphal growth, with the fungus consuming the larva from the inside out [14]. Two widespread pathogenic species, *Ascosphaera aggregata* and *A. apis*, are of economic interest because of their potential to negatively impact populations of commercial pollinators, namely *Apis mellifera* L. and *Megachile rotundata* (Fabricius) [15], [16].

Although *Ascosphaera* lives in association with both solitary and social bees the majority of species (25 out of 28) were originally described from solitary bees. Within the nests of solitary bees *Ascosphaera* grows on pollen provisions where an egg has failed to develop, on larval feces, on the surface of cocoons, within larvae, and on the diverse materials used by different bee species for brood cell construction [4], [5], [6]. Unlike their social relatives (e.g., honey bees), solitary bees lack adult-larva interaction, there is no nursing of the brood and no cooperative behavior (including social immunity) [17]. A consequence of no adult-larva contact and no nursing is that the brood is mass provisioned rather than progressively provisioned like their social counterparts; this means that once an egg hatches the larva has all the food it will need to complete development into an adult [17]. Following their flight and nesting period solitary bees overwinter in their individual brood cells with no activity until emergence the following spring or early summer. In this way solitary bee nests provide a relatively stable, undisturbed micro-environment that appears suitable for the growth of these specialised fungi.

The only monographic work on *Ascosphaera* [5] focused on collections from an important commercial pollinator in Canada, the alfalfa leafcutting bee *Megachile rotundata*. While limited in scope, this monograph, which included the first key to the genus, remains the most useful and comprehensive reference for the identification of *Ascosphaera* species. Given the importance of wild pollinators and their increasing role in buffering the loss of honeybee pollination services [18] a more complete monograph with an updated key to these bee-specialist fungi is much needed.

Seven of the 28 described species of *Ascosphaera* are currently known from Europe. Here we describe a new species from Denmark occurring in the nests of the wild solitary bee *Chelostoma florisomne* L. To stimulate interest and to facilitate the identification of *Ascosphaera* species so far known from Europe, we provide a key and descriptions for these species. Cumulative host reports and species distributions are also included with the hope that this information will result in additional records for these under-collected fungi.

## Materials and Methods

### Morphological study

Descriptions of spore cysts and ascospores were made from observations of spore cysts mounted in water on a glass slide. Measurements and light photomicrographs were made on an Olympus AX70 Provis light microscope and Olympus SZX16 dissecting microscope. Herbarium acronyms follow those of Index Herbariorum [19].

### Culture and isolation

Attempts to isolate and culture the fungus were made by placing spore cysts and hyphae on three different solid agar media: malt agar with 20% dextrose (MY20), V8® agar with 2% yeast extract (V8YE), and malt extract agar (MEA). To induce spore germination spore suspensions were prepared from spore cysts placed in a modified V8 spore germination broth [20] and exposed to CO_2_ as described in Wynns et al. [13].

### Molecular study

Genomic DNA was obtained by plucking 5–10 spore cysts and grinding them inside a 1.5 ml Eppendorf tube. DNA was isolated using the Qiagen DNeasy Plant Mini Kit (Hilden, Germany) using the standard protocol and eluted in two separate 50–100 µl fractions to avoid over-dilution.

We sequenced the entire nuclear ribosomal ITS region (ITS1-5.8S-ITS2) for *A. callicarpa* sp. nov. Genomic DNA was amplified using ITS1F and ITS4 primers [21]. PCR reactions were prepared for a final 50 µl volume containing 29.8 µl of sterile deionized water, 5 µl of *Taq* polymerase reaction buffer (Sigma®), 1.0 µl 10 mM dNTPs, 3.0 µl 25 mM MgCl_2_, 0.2 µl *Taq* DNA polymerase (Sigma®), 5.0 µl each 10 µM primer and 1 µl of genomic DNA template. PCR was performed on a Biometra® thermocycler (Whatman) under the following conditions: step 1) 1 min at 95 C, 2) 45 sec at 95 C, 3) 40 sec at 52 C, 4) 1 min 30 sec at 72 C, 5) return to step 2 30 times, 6) final step of 10 min at 72 C. Samples were kept at 4 C until electrophoresis was performed on a 1% agarose TAE gel and visualized with EZvision One® (Amresco). PCR reactions were cleaned using Qiaquick® PCR purification kit (Qiagen) and sent to Eurofins MWG Operon AG (Ebersberg, Germany) for sequencing. The nucleotide sequence was assembled using BioEdit [22] and subjected to a BLASTn search in GenBank.

### Nomenclature

The electronic version of this article in Portable Document Format (PDF) in a work with an ISSN or ISBN will represent a published work according to the International Code of Nomenclature for algae, fungi, and plants, and hence the new names contained in the electronic publication of a PLOS ONE article are effectively published under that Code from the electronic edition alone, so there is no longer any need to provide printed copies. In addition, new names contained in this work have been submitted to MycoBank from where they will be made available to the Global Names Index. The unique MycoBank number can be resolved and the associated information viewed through any standard web browser by appending the MycoBank number contained in this publication to the prefix http://www.mycobank.org/MB/. The online version of this work is archived and available from the following digital repositories: PubMed Central, LOCKSS.

## Results and Discussion

### Culture and isolation

Despite repeated attempts, we were unable to obtain in vitro mycelial growth or induce ascospore germination of *Ascosphaera callicarpa*.

### Molecular study

An ITS sequence was obtained for *A. callicarpa* (GenBank accession: JX070046). A BLASTn search of the ITS sequence revealed a highest sequence-similarity to other *Ascosphaera* species.

### Key to European species of *Ascosphaera*

**Figure 1:**
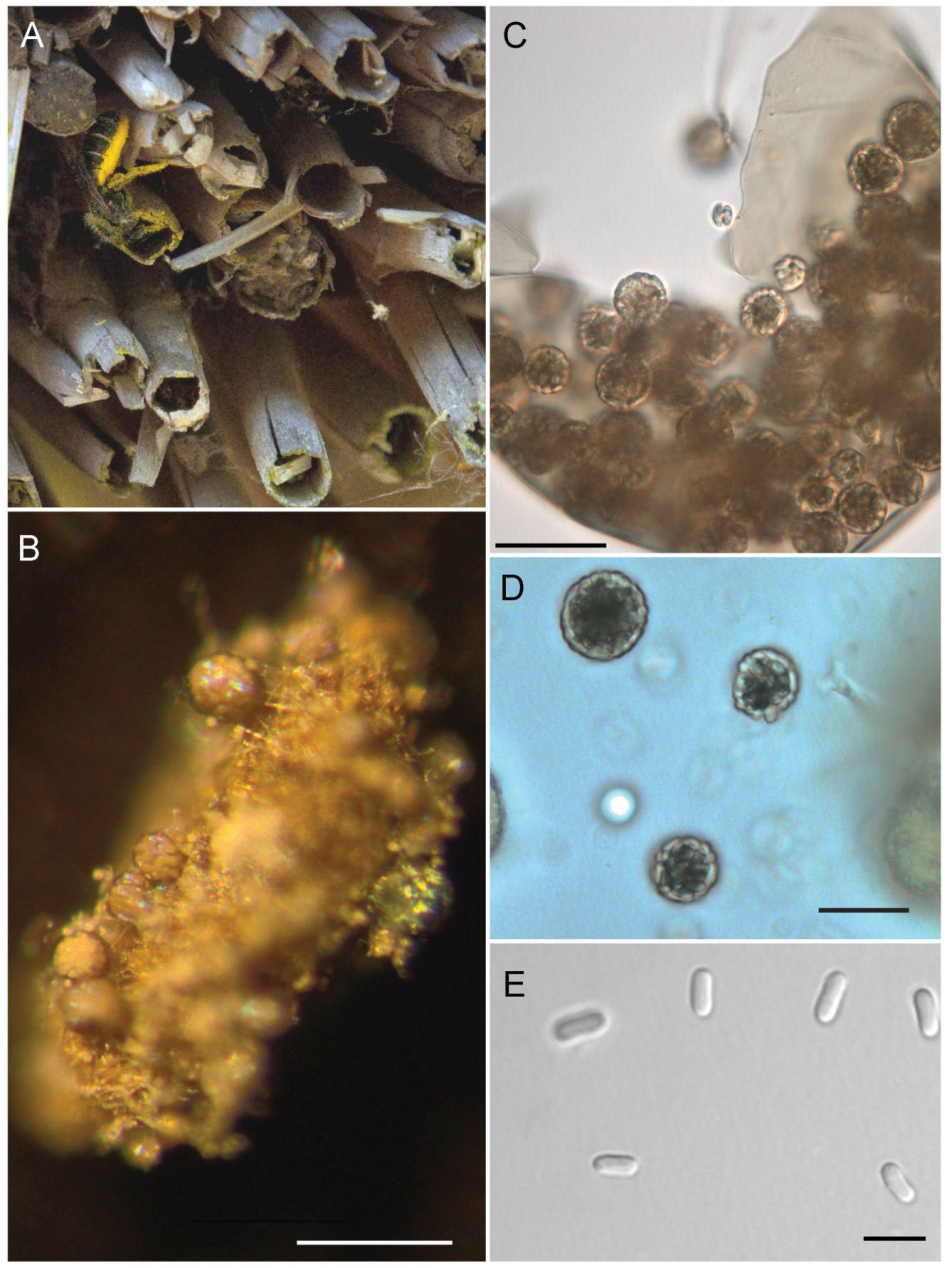
Ascosphaera callicarpa. A) habitat. *Phragmites* reeds and female *Chelostoma florisomne* returning with pollen for her brood. B) fecal pellet of *C. florisomne* larva covered with spore cysts; pale spore balls are visible through the transparent spore cyst wall. C) close-up of spore cyst showing spore balls and smooth, unornamented spore cyst wall. D) spore balls. E) bacilliform ascospores. B, photographed from *A.A. Wynns 5168*; C–E from *A.A. Wynns 5166*. Scale bars: B = 200 µm, C = 50 µm, C = 10 µm, D = 15 µm, E = 10 µm.

**Figure 2:**
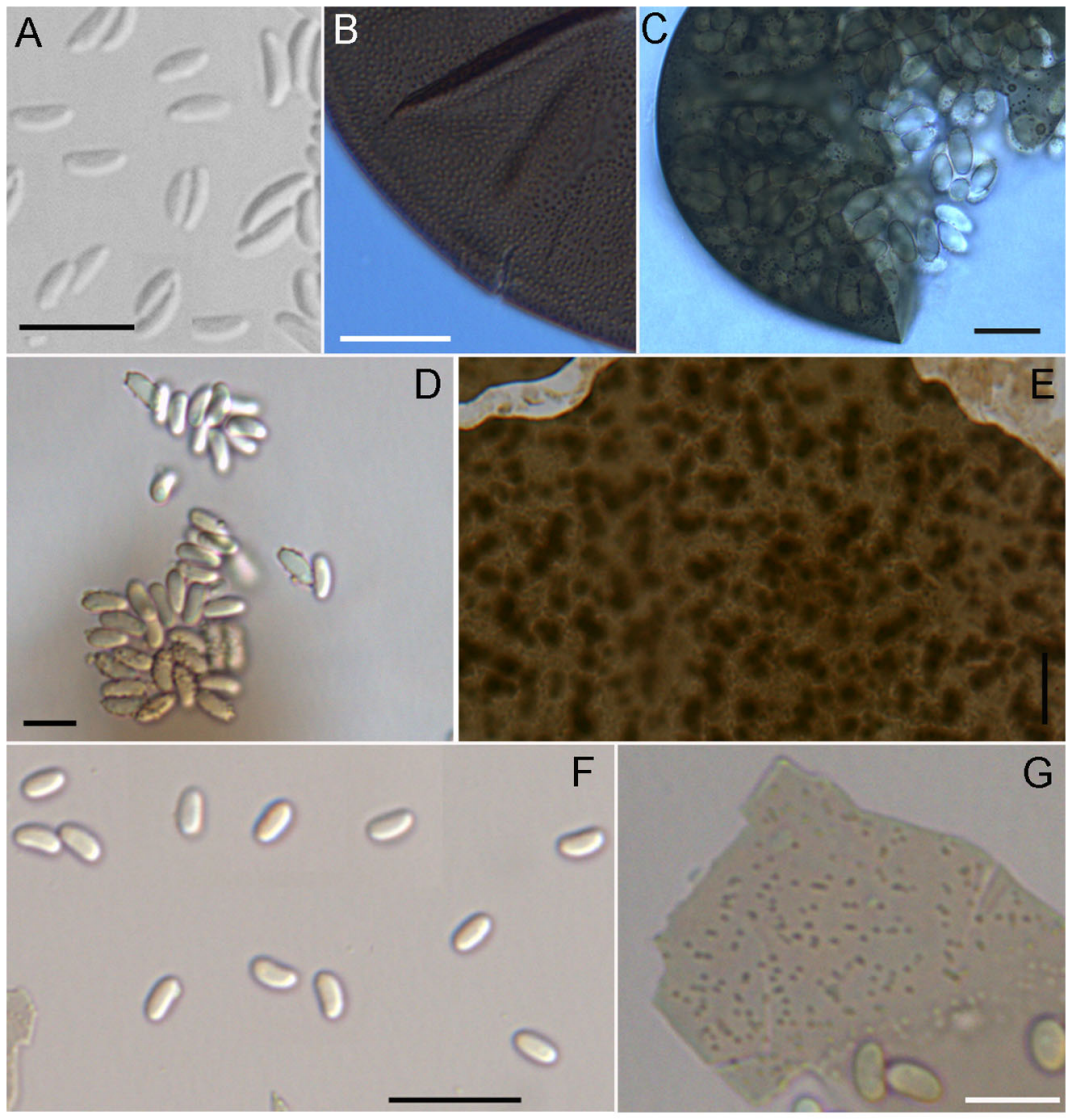
Light microphotographs of *Ascosphaera tenax, A. atra*, *A. major*, and *A. apis*. *Ascosphaera tenax* A) ascospores. B) punctate spore cyst wall.; *A. atra* C) broken spore cyst with ascospores.; *A. major* D) ascospores with attached granules. E) close-up of spore cyst wall.; *A. apis* F) ascospores. G) detail of pale spore cyst wall with minute spots. A–B photographed from holotype; C from ARSEF 693; D from *A.A. Wynns 5170*; E from *A.A. Wynns 5175*; F from *A.A. Wynns 5174*. Scale bars A = 5 µm, B–C = 10 µm, D = 5 µm, E–F = 10 µm.

**Figure 3:**
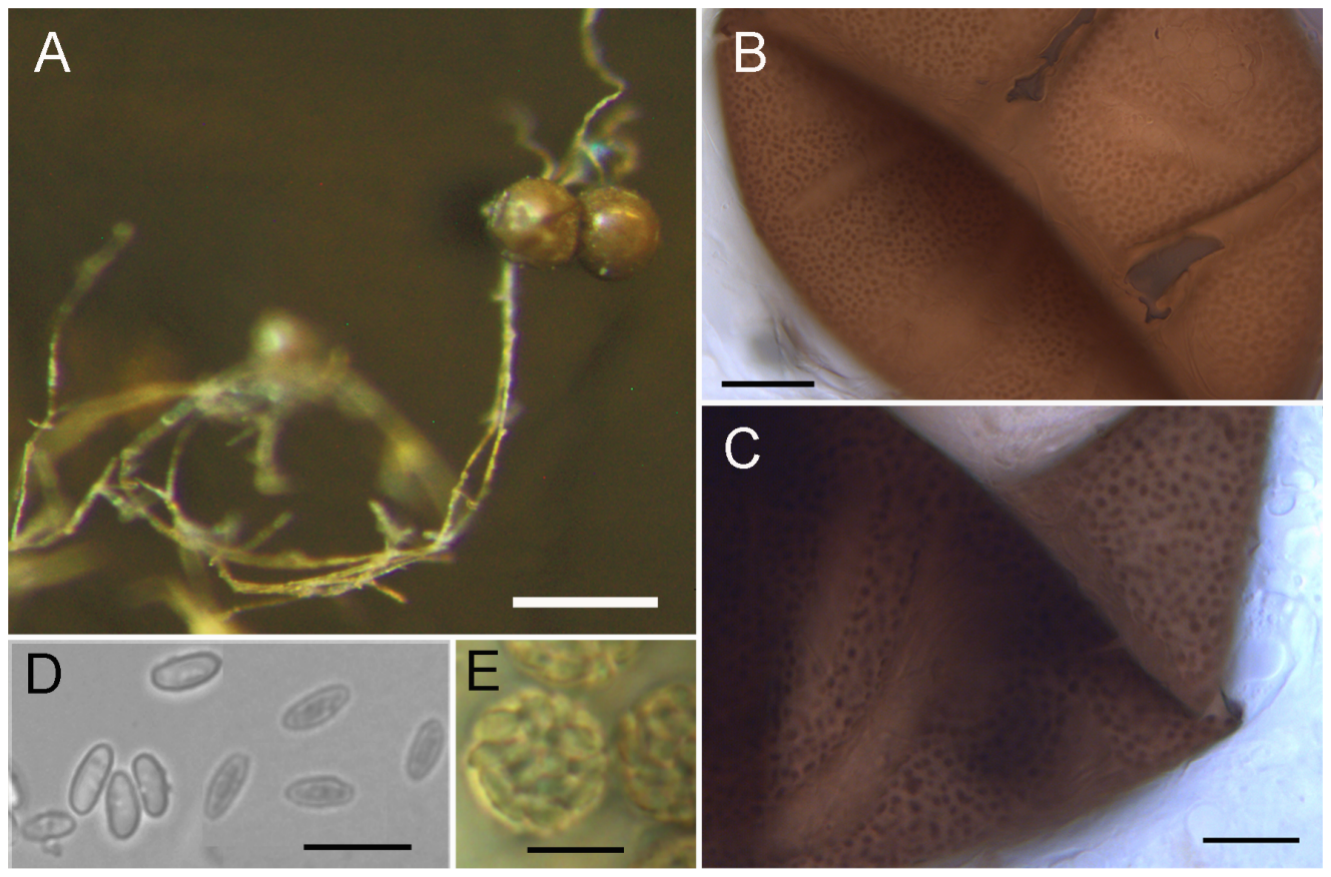
Light microphotographs of *Ascosphaera fimicola*. A) two opaque, iridescent spore cysts still attached to hyphae. B–C) close-up of spore cyst showing maculate wall, D) ellipsoid ascospores with a few small granules attached to their surface. E) spore ball. A, photographed from *A.A. Wynns 5167*; B–C, E from *A.A. Wynns 5130*; D from *J.P. Skou* s.n. (paratype). Scale bars: A = 500 µm, B = 20 µm, C = 10 µm, D = 5, µm E = 15 µm.

**Figure 4:**
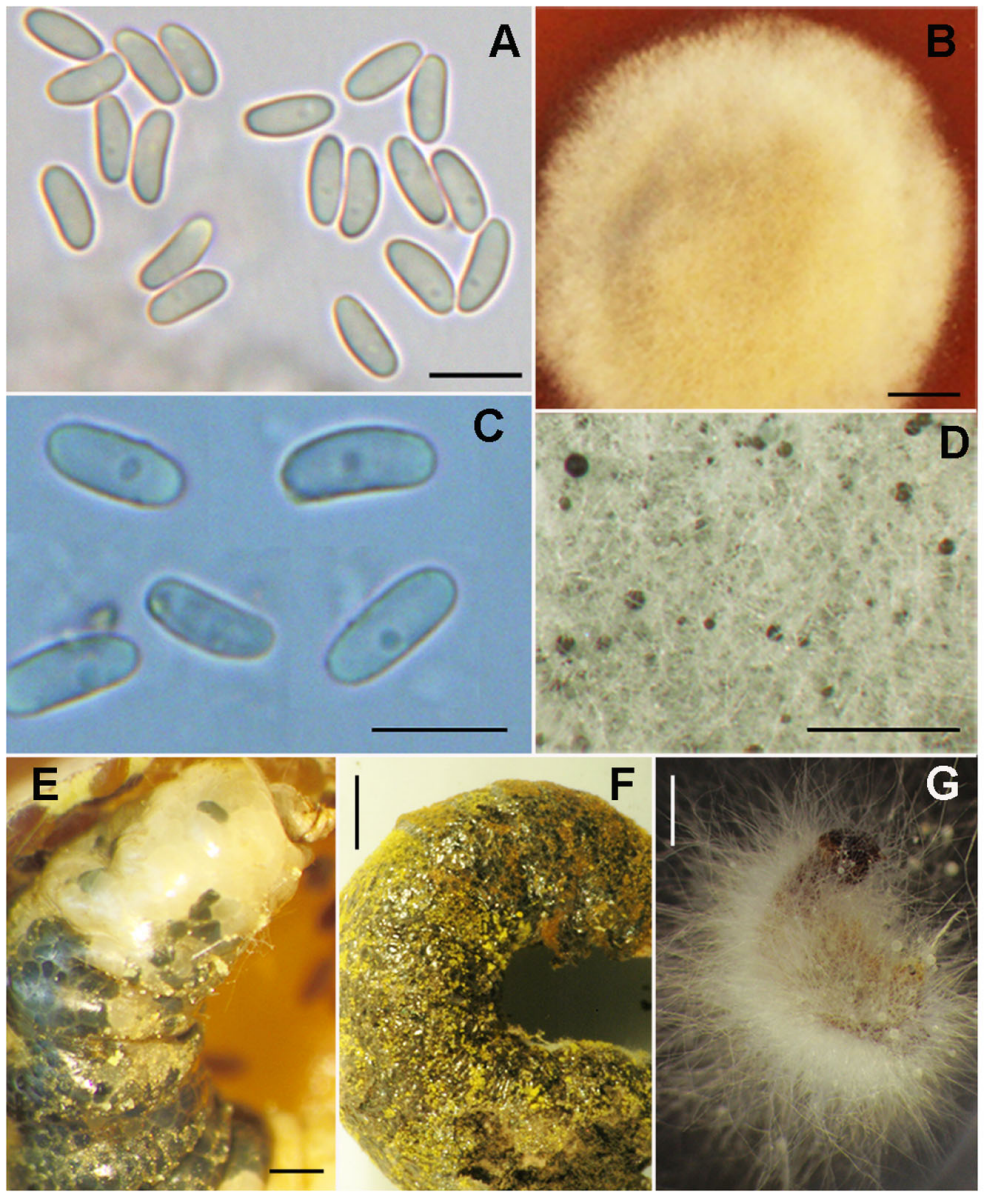
Light microphotographs of *Ascosphaera aggregata* and *A. proliperda*. *Ascosphaera aggregata* A) ascopores. B) culture on V8 agar medium showing pale buff mycelium.; *A. proliperda* C) ascospores. D) culture on SDA showing white mycelium and scattered black spore cysts. E–F) bee larvae with chalkbrood caused by *A. aggregata*. E) fresh *Chelostoma florisomne* cadaver showing mature (black) and immature (white) spore cysts below the cuticle. F) dry *Osmia bicornis* larva swollen from fungal growth and spore cyst production below the cuticle. G) aerial hyphae and spore cysts on bee larva with chalkbrood caused by *A. proliperda*. A, photographed from *A.A. Wynns 5144*; B from *A.A. Wynns 5162*; C from *A.A. Wynns 5055*. Scale bars: A = 10 µm, B = 2 cm, C = 5 µm, D = 825 µm, E = 2 mm, F = 1 mm, G = 2 mm.

1. Wall of spore cyst smooth; ascospores cylindrical with rounded ends, (3.1–) 4.0×1.6(−2.0) µm; saprotroph*A. callicarpa* A.A. Wynns (Fig. 1)

1. Wall of spore cyst with minute or conspicuous dark spots; ascospores not cylindrical; saprotroph or pathogen2

 2. Ascospores broadly sub-falcate, with a tendency to be trigonal when view on-end, 1.9–3.5×0.6–0.9 µm; saprotroph*A. tenax* Skou (Figs. 2A–B)

 2. Ascospores not sub-falcate or trigonal in cross section3

3. Ascospores always >2 µm wide, 4–7.9×2.3–6.5 µm, ellipsoid to broadly ellipsoid; spore balls not persistent; saprotroph*A. atra* Skou & K. Hackett (Fig. 2C)

3. Ascospores otherwise; spore balls persistent; pathogen or saprotroph4

 4. Spore cysts not exceeding 125 µm diameter5

 4. Spore cysts mostly exceeding 125 µm diameter6

5. Ascospores 3.0–5.0×1.3–1.8 µm; at least some ascospores and spore balls with attached granules; spore cyst wall brown with small spots visible at low magnification; saprotroph*A. fimicola* Skou (Fig. 3)

5. Ascospores 2.1–3.9×1.1–1.7 µm; ascospores and spore balls always without granules; spore cyst wall pale greenish to yellowish brown, with nearly smooth walls, minute spots visible at high magnification; obligate parasite, cause of chalkbrood disease of honeybees*A. apis* (Maasen ex Claussen) L.S. Olive & Spiltoir (Figs. 2F–G)

 6. Ascomata often ≥400 µm diameter, forming a dense layer beneath the cuticle of bee larvaewith chalkbrood disease; ascospores 3.4–5.9×1.3–2.6 µm, ellipsoid, sub-cylindrical or allantoid; obligate pathogen*A. aggregata* Skou (Figs. 4A–B, E–F)

 6. Spore cysts mostly less than and not exceeding 400 µm in diameter, developing on aerial hyphae above the cuticle of larvae with chalkbrood disease or growing saprotrophically on the cocoon, feces or leaf lining of a brood cell7

7. Ascospores (2.4–) 2.8–4.0 (−5.0)×1.0–1.8 (−2.0) µm*A. major* (Prökschl & Zobl) Skou (Figs. 2D–E)

7. Ascospores 3.5–6.5×1.7–3.5 µm*A. proliperda* Skou (Figs. 4C–D,G)

### Taxonomy

***Ascosphaera aggregata*** Skou, Friesia 11: 64, 1975.

Type: DENMARK.ThurØ, on larvae of *Osmia rufa* L., *J.P. Skou* s.n. (holotype, c!).

**Figs. 4A–B, E–F** **.**

#### Description

Mating system unknown but possibly homothallic [4]. Pathogenic. Infected larvae swollen, black, and filled with a solid core of pale buff mycelium. Ascomata black to dark brown spore cysts produced below surface of larval cuticle in a crowded continuous layer [23] or scattered and appearing as small individual boils [24], 280–750×130–290 µm, spherical or conical and faceted from being tightly packed beneath the larval cuticle; wall light reddish brown to black, minutely punctate. Spore balls pale brown to yellowish brown with small brown granules attached to surface, 9–25 µm diameter, mostly persistent. Ascospores ellipsoid to sub-cylindrical or allantoid, 3.4–5.9×1.3–2.6 µm. Culture on V8YE with moderate growth after 14 days, low, pale buff with a darker, brownish centrum with age, occasionally producing nutriocytes on aerial hyphae (A.A.Wynns pers. obs.) and immature spore cysts below agar surface [4].

#### Ecology and distribution

*Ascosphaera aggregata* is an obligate pathogen with a preference for bees belonging to the family Megachilidae. This species has a broad distribution, with reports from both North America and Europe. Like *A. apis*, the distribution of *A. aggregata* is probably closely tied to the exchange and transport of bees (e.g. *M. rotundata*) for the pollination of commercial crops. In Europe *A. aggregata* is known from Denmark [23], Germany [present study], Spain [23], [25] and Sweden [present study]. Attempts to isolate and grow *A. aggregata* in culture often results in the co-isolation of another pathogenic species, *A. proliperda*. Although *A. proliperda* and *A. aggregata* can be difficult to separate based on microscopic morphological features their growth in culture is strikingly different (Figs. 4B,D; see also *A. proliperda* for further discussion on its co-occurrence with *A. aggregata*).

#### Additional specimens examined

CANADA. Alberta: Brooks, on larvae of *M. rotundata*, 1988, J Jakobsen s.n. (c). DENMARK. Thurø: Svendborg Kommune, on *Osmia rufa* ( = *O. bicornis* L.), 1974, *J.P. Skou* s.n., paratype (c); On *O. rufa*, 1974, *J.P. Skou* s.n., holotype, (c); Zealand: Frederikssund Kommune, Slangerup, organic apple orchard, on larva of *O. rufa*, 2010, *A.A. Wynns 5152*; Roskilde Kommune, Roskilde, on larva of *O. rufa*, 2010, *A.A. Wynns 5144*; Taastrup Kommune, Taastrup, Højbakkegård, on larvae of *O. rufa*, 2010, *A.A. Wynns 5145* (c). USA. Nevada: On *Megachile pacifica* (Panzer) ( = *M. rotundata*), 1975, K. Hackett s.n. paratype (c). SPAIN: On *M. rotundata*, 1972, *J.P. Skou* s.n., paratype (c); on *M. rotundata*, 1973, *J.P. Skou* s.n. paratype (c).

***Ascosphaera apis*** (Maasen ex Claussen) L.S. Olive & Spiltoir in Spiltoir & Olive, Mycologia 47: 242, 1955.

**≡** *Pericystis apis* Maasen ex Claussen, Mitt. biol. BundAnst. Ld-u. Forstw.10: 470. 1921.

**Figs. 2F–G**

#### Description

Mating system heterothallic. Pathogenic. Infected larvae shrunken, pale buff, covered by a weft of hyphae, with or without the production of ascomata. Ascomata greenish (immature) to black (mature) spore cysts produced on aerial hyphae above the larval cuticle, 40–119 µm in diameter; wall pale greenish to yellowish-brown, nearly smooth with minute punctae at high magnification. Spore balls hyaline to pale yellowish, without granules, 7–20 µm in diameter, mostly persistent. Ascospores ellipsoid to sub-allantoid, 2.1–3.9×1.1–1.7 µm. Culture on SDA with rapid growth after 2–6 days, white with abundant production of spore cysts when both mating strains are present.

See Skou [7], Bissett [5], and Aronstein & Murray [26] for additional descriptions.

#### Ecology and distribution

*Ascosphaera apis* is an opportunistic pathogen of honeybees. Experimental trials showed *A. apis* is able to induce chalkbrood in the solitary bee *M. rotundata* [14]; however, *A. apis* is not known to live in association with solitary bees in nature. Reports of chalkbrood caused by *A. apis* in solitary bees before 1972 are most likely attributable to pathogenic species described after this time; e.g., *A. major*, *A. aggregata* or *A. proliperda* (the later two species are pathogens specific to solitary bees). Originally described from Germany, *A. apis*, is now known from all continents where honeybees are kept.

Additional specimens examined. USA. Texas: Weslaco, 26 Jun 2003, *K.D. Murray* s.n., ARSEF 7405 (+), 7406 (−).

***Ascosphaera atra*** Skou & K. Hackett, Friesia 11: 279, 1979.

Type: U.S.A. Nevada, isolated from larva of *Megachile pacifica* with ragged-brood disease, *36836* (c), CBS 524.75 (holotype, c!).

**Fig. 2C**

#### Description

Mating system homothallic. Ascomata black, globose spore cysts, 30–140 µm; wall dark brown, punctate, punctae appearing as uniform dark circles often of variable size. Spore balls hyaline to pale yellowish brown, 8–17 µm diameter, evanescent. Ascospores ellipsoid to broadly ellipsoid, 4–7.9×2.3–6.5 µm, with or without small granules attached to the surface of the spore wall. Culture on SDA with moderate growth after 7 days, white to greyish-buff with abundant production of black spore cysts on aerial hyphae and on hyphae growing beneath the surface of the agar.

#### Ecology and distribution

*Ascosphaera atra* is a fast-growing saprotroph associated primarily with solitary bees. This species is typically found growing on pollen provisions. Less common substrates from which *A. atra* has been isolated include the surface of a diseased *M. rotundata* larva with chalkbrood caused by *A. aggregata* [27], from pollen within the gut of an otherwise healthy *M. rotundata* larva [7] and from the honey of *A. mellifera* [4]. *Ascosphaera atra* is the only species of the genus that has been found growing on plant material (grass silage) outside of the bee habitat [6]. Pathogenicity studies [14], [27] demonstrated that *A. atra* is not a pathogen of solitary bees; however, Vojvodic et al. [28] concluded that it is a weak pathogen of honeybees. More work is needed to determine if *A. atra* is comparable to some of its bee- pathogen congeners e.g. *A. aggregata* and *A. apis*. The perceived pathogenicity of this species in honey bee larvae may be more closely tied to its rapid growth on suitable substrates. *Ascosphaera atra* is the most extensively studied saprotrophic species of *Ascosphaera*. This is reflected in the multiple reports from N. America [4], [14], Europe [[6], present study], New Zealand and Australia [4].

Additional specimens examined. *Ascosphaera atra*. AUSTRALIA. Peel: Waroona, *A. mellifera* honey, Nov 1994, *D.L. Anderson 198*, ARSEF 5147. CANADA. Alberta: Beaverlodge, Peace River region, from pollen in *M. rotundata* cells, Jan 1985, *D. Farney* s.n., DAOM 188981. USA. Oregon: Ontario. *M. rotundata*, Jun 1979, *J.D. Vandenberg 6*, ARSEF 693.

***Ascosphaera callicarpa*** A.A. Wynns, sp. nov. [urn:lsid:indexfungorum.org:names:518624]

Type. DENMARK. Zealand: Lejre Kommune, Sagnlandet (“Land of Legends”) Lejre, Landbohusene, on fecal pellets of *Chelostoma florisomne* nesting in the *Phragmites* reeds of thatched roof of shed behind 19^th^ century cottage, 55°37′11″N; 11°22′13″, 2010, *A.A.Wynns 5165* (holotype, c).

**Fig. 1**

#### Description

Mating system unknown. Ascomata pale brown, semi-transparent and somewhat iridescent spore cysts (Fig. 1B), globose to subglobose 64–101 µm in diameter; wall smooth (Fig. 1C). Spore balls 10–16 µm in diameter, center grayish–brown to colorless, ascospores arranged spirally or not (Fig. 1C, D). Ascospores bacilliform, (3.1–)4.0×1.6(−2.0) µm, colorless or slightly brownish (Fig. 1D–E); no attached granules. Mycelium sparse, white. No growth in culture on MY20, V8YE or MEA; no spore germination in V8 spore germination broth, either with or without the addition of carbon dioxide.

#### Ecology and distribution

Common in the nest reeds of the solitary bee *C. florisomne* where it grows on the fecal pellets of this bee. Although not definitely known, the distribution of *A. callicarpa* is probably closely tied to that of *C. florisomne*. This fungus was not found in association with other bees, e.g. *Osmia* and *Megachile*, although these bees were observed nesting in the same *Phragmites* reeds as *C. florisomne. Ascosphaera callicarpa* appears to be solely saprotrophic; it was not found in association with diseased bees or where a larva had failed to develop. *Ascosphaera callicarpa* is so far known only from the island of Zealand, Denmark.

#### Etymology

The epithet *callicarpa* means with beautiful fruits, here referring to the spore cysts.

#### Preliminary conservation status

*Ascosphaera callicarpa* should be sought in other aggregations of *C. florisomne* in thatched roofs throughout Europe in order to assess its conservation status. As a possible obligate associate of the bee *C. florisomne*, the conservation of this fungus should be considered dependent on the conservation of its host.

#### Additional specimens examined

DENMARK. Zealand: Lejre Kommune, Sagnlandet Lejre, Landbohusene, shed behind 19^th^ century houses. All specimens on fecal pellets of *C. florisomne* nesting in the *Phragmites* reeds of the thatched roof, 2008, *A.A.Wynns 5011*, *5012*, *5013*, *5014*, *5015*, *5018*, *5025*, *5026*, *5027*, *5072*, *5074*, *5136*, all specimens in c; 2011, *A.A.Wynns 5166*, *5168* (c); Sorø Kommune, Kristiansminde, University of Copenhagen field station, east facing side of classroom building, growing on the fecal pellets of *C. florisomne* nesting in *Phragmites* reeds of the thatched roof, 2012, *A.A. Wynns 5169*, *5170* (c).

#### Morphological comparison of *A. callicarpa* with *A. fimicola*

*Ascosphaera callicarpa* most closely resembles *A. fimicola* Skou which also grows on the fecal pellets of bees. This new species is distinguishable from *A. fimicola* by a pale brown, highly transparent fragile spore cyst (Fig. 1B–C) with a wall (Fig. 1C) that is not sculptured or maculate as in *A. fimicola* (Fig. 3B–C). The spore cysts of *A. fimicola* (Fig. 3A) are dark brown to pale brown, also somewhat iridescent, and if transparent, not as strikingly so as in *A. callicarpa* (Fig. 1B). The spores of *A. fimicola* are ellipsoid-fusiform (Fig. 3D) and often have small brown granules attached to their surface while the spores of *A. callicarpa* are bacilliform (Fig. 1E) without surface granules. *Ascosphaera callicarpa* grows on digested *Ranunculus* pollen voided by *C. florisomne*. It is not clear if *A. callicarpa* grows on pollen collected from other plants since *C. florisomne* is strictly oligolectic on *Ranunculus* species [29].

***Ascosphaera fimicola*** Skou, Friesia 11: 68, 1975.

Type: DENMARK, ThurØ, on fecal pellets from larvae of *Osmia rufa*, *J.P. Skou* s. n., (holotype, c!).

**Fig. 3**

#### Description

Mating system not known. Ascomata light to dark brown, somewhat iridescent, glistening spore cysts, (25–) 64–125 µm in diameter; wall brown, punctate, punctae minute and of uniform in size. Spore balls yellowish, with small granules on the surface, (5–)10–15(−20) µm in diameter, mostly persistent. Ascospores ellipsoid to sub-allantoid, 3.0–5.0×1.3–1.8 µm with or without small granules attached to the spore wall. Mycelium on natural substrate noticeable, stringy, white and opaque. No growth in culture.

#### Ecology and distribution

*Ascosphaera fimicola* grows saprotrophically on the larval feces and cocoons of the solitary bee *Osmia bicornis* (syn. *O. rufa*) and was recently collected on the larval feces of *Cacoxenus indagator* (Diptera: Drosophilidae) a cleptoparasite of this bee. Despite extensive collecting, *A. fimicola* was not found on the larval feces of the solitary bee *C. florisomne*. The composition of the pollen provisions of these bees may play a role in the absence or presence of *A. fimicola* in their nests. *Chelostoma florisomne* feeds exclusively on pollen from the plant genus *Ranunculus* (Ranunculaceae) [29] while *O. bicornis* often collects pollen from the plant family Rosaceae [30]. The last report of *A. fimicola* prior to our study was in 1975 [23]. We found that this species is more common than the previous few collections indicate. The known distribution of *A. fimicola* is restricted to Denmark but, like other species in the genus, this narrow distribution is most likely an artifact of under-collecting because of a more focused interest in the pathogens rather than the saprotrophs.

#### Additional specimens examined

DENMARK. Zealand: Taastrup Kommune, Højbakkegård Allé 3, on feces of *Cacoxenus indagator* in *O. rufa* brood cell, 2008, *A.A. Wynns 5123* (c); on feces and cocoon of *O. rufa*, 2010, *A.A. Wynns 5147*, *5167* (c). Frederikssund Kommune, Slangerup, residence and farm of Verner Andersen, on pollen and feces in nest cell of *O. rufa*, 2010, *A.A. Wynns 5149*, *5151* (c). langeland: Langeland Kommune, Rudkøbing, Skovsgaard, Kågårdsvej 12, on cocoon of *O. rufa* and *C. indagator*, 2008, *A.A. Wynns 5130* (c); on cocoon and feces of *O. rufa*, 2008, *A.A. Wynns 5131* (c). THURØ: Svendborg Kommune, on fecal pellets of *O. rufa*, 1972, *J.P. Skou* s.n., paratype (c).

***Ascosphaera major*** (Prökschl & Zobl) Skou, Friesia 10:15, 1972.

type: DENMARK. Zealand: Glostrup, isolated from chalkbrood cells of *Megachile centuncularis*, cbs 686.71 (neotype, cbs h-9050, non vidi).

≡*Pericystis apis* Maasen ex Claussen var. *major* Prökschl & Zobl in Prökschl, Arch. Microbiol. 18: 200. 1953.

≡*Ascosphaera apis* (Maasen ex Claussen) L.S. Olive & Spiltoir var. *major* (Prökschl & Zobl) L.S. Olive & Spiltoir in Spiltoir & Olive, Mycologia 47: 243. 1955.

**Figs. 2D–E**

#### Description

Mating system heterothallic. Ascomata dark brown to black, spore cysts, 60–150(−380) µm in diameter; wall greenish brown, with indistinct puntcae or small granules attached to the inner surface, occasionally with larger crystalliferous brown precipitations with age. Spore balls hyaline to greyish-brown, (6–)14–18(−24) µm in diameter, usually with granules attached to the surface. Ascospores suballantoid or bacilliform. (2.4–)2.8–4.0(−5.0)×1.0–1.8(−2.0) µm, at least some with small granules attached to the spore wall. Mycelium white to greyish-white. Culture on V8YE with moderate growth after 10 days, with abundant production of spore cysts when both strains are present.

#### Ecology and distribution

*Ascosphaera major* causes chalkbrood in *Apis mellifera* [31], [32] and *Megachile centuncularis* [7]. It is more often found growing saprotrophically on larval feces within the brood cells of *M. centuncularis* [7]. In the present study *A. major* was found growing on the larval feces and leaf material lining the brood cell of a species of *Megachile* and on the larval feces and pollen provisions of *O. bicornis*. The frequency of *A. major* as a cause of chalkbrood in honeybees is not known. Outwardly *A. apis* and *A. major* induce the same disease symptoms; therefore, the etiology of chalkbrood in honeybees should be carefully verified by morphological study of the fungus to distinguish infections by *A. apis* or *A. major* or to identify co-infection with both species. *Ascosphaera major* is known from N. America [33], [34] and Europe. In Europe this species is reported from Switzerland [32], Austria [31] and Denmark [7], [9].

#### Additional specimens examined

DENMARK. Zealand: Frederikssund Kommune, Slangerup, organic apple orchard belonging to Verner Andersen, growing on pollen and feces of *O. bicornis*, 2010, *A.A. Wynns 5150* (c); Lejre Kommune, Lejre Forsøgscenter, growing on cocoon and between walls of leaf lining of healthy *Megachile* sp., 2008, *A.A. Wynns 5038* (c); Roskilde Kommune, Roskilde, Gøderupvej 5, on leaf-lining of brood cell belonging to *Megachile* sp., 2010, *A.A. Wynns 5173*, on larval feces of *Megachile* sp. without disease, 2010, *A.A. Wynns 5175*.

***Ascosphaera proliperda*** Skou, Friesia 10: 15. 1972.

type: DENMARK. Zealand: Frederiksberg Kommune, Frederiksberg, in *Megachile centuncularis* larvae collected from the greenhouse of the Royal Veterinary and Agricultural University, *J.P. Skou* s.n., Jun 1967, CBS 687.71 (holotype, cbs h-6723, non vidi).

**Figs. 4C–D,G**

#### Description

Mating system homothallic [5]. Pathogenic. Infected larvae shrunken, covered by erect or low compact aerial hyphae bearing ascomata. Ascomata black spore cysts produced on tips of aerial hyphae above the larval cuticle, 60–250(−400) µm in diameter; wall dark-brown, appearing mottled from the confluence of very fine granules on the inner surface. Spore balls pale brown to sub-hyaline, 9–17(−25) µm, often with small brown granules on the surface. Ascospores sub-cylindrical or sub-allantoid, 3.5–6.5×1.7–3.5 µm, hyaline to sub-hyaline, with or without minute granules attached to the surface. Culture on MY20 with rapid growth after 7 days, white with abundant production of spore cysts.

#### Ecology and distribution

*Ascosphaera proliperda* causes chalkbrood in *Megachile centuncularis* [9], *M. rotundata* [35] and *O. bicornis* [present study]. *Ascosphaera proliperda* has repeatedly been isolated from surface sterilized chalkbrood cadavers of *M. rotundata* and *O. bicornis* infected with *A. aggregata* [15], [35]. Interestingly, these cadavers exhibit typical symptoms for *A. aggregata* infection i.e. the cuticle of the host is intact with spore cysts just below the cuticle rather than above the cuticle as is typical for *A. proliperda*. The co-occurrence of *A. proliperda* and *A. aggregata* in chalkbrood larvae adds to the difficulty of isolating *A. aggregata* since the former species is much faster growing. Unless isolation or PCR diagnostics (see [36]) are attempted, *A. proliperda* can be easily overlooked in chalkbrood cadavers where *A. aggregata* is the predominant fungus. *Ascosphaera proliperda* is known from Europe [9] and N. America [35]. This species may prove to be more widespread if possible co-infections with *A. aggregata* in chalkbrood larvae are taken into consideration.

#### Additional specimens examined

GERMANY. Isolated from the surface of a sterilized *A. aggregata* chalkbrood cadaver of *O. bicornis*, 2010, leg. T. Conrad, *A.A. Wynns 5055* (c).

***Ascosphaera tenax*** Skou & S.N. Holm, *Mycotaxon* 35: 212, 1989.

Type: DENMARK, Nekselø: Kalundborg Kommune, inside cocoons of *Megachile willughbiella*, 1985, *J.P. Skou* s.n., (holotype, c!).

**Figs. 2A–B**

#### Description

Mating system unknown. Ascomata lustrous black, less often dark brown, spore cysts, (33–)40–90(−105) µm diameter; wall dark brown,1.5 µm thick, tough and leathery, smooth or minutely punctate. Spore balls hyaline, (7.7–)9–14(−15.4) µm diameter. Ascospores sub-falcate, with a tendency to be trigonal when viewed on-end, 1.9–3.5×0.6–0.9 µm.

#### Ecology and distribution

*Ascosphaera tenax* grows saprotrophically on pollen provisions, larval feces and the inner side of cocoons of *Megachile willughbiella* and *M. rotundata*. Spore cysts are common beneath the inside of the leaf cap of *Megachile* cells. The last collections of *A. tenax* date from 1988, when the species was found growing in nearly half (18 out of 44) *M. willughbiella* cocoons examined [24]. *Ascosphaera tenax* is known only from Denmark on the islands of Nekselø and Zealand. More focused collecting is needed to determine its real geographical range.

## Conclusion

Our study is the first to provide a regional key to *Ascosphaera*. With the addition of *A. callicarpa* sp. nov., eight *Ascosphaera* species are now known from Europe (Table 1). Our collections of *A. fimicola* (see discussion under *A. fimicola*) from a dipteran cleptoparasite of *Osmia bicornis* add to the mounting evidence that, although undoubtedly a bee specialist, *Ascosphaera* is not restricted to bees; further evidence includes an isolated report of the saprotroph *A. atra* growing on grass [6] and molecular based identification of *Ascosphaera* DNA from *Eristalis* (Diptera: Syrphidae) and *Vespula* (Hymenoptera: Vespidae) species [37]. As previously suggested by Wynns [42], *Ascosphaera* should be sought outside the bee habitat in association with other pollenivorous insects and where high-sugar substrates are available. Reports of *Ascosphaera* in non-apoidean insects are quite possibly relevant for the control of chalkbrood in commercial bee pollinators since these insects may act as pathogen reservoirs or vectors of *Ascosphaera*. More frequent collections of *Ascosphaera* are needed to begin to grasp the diversity and ecology of these fungi in nature and to elucidate their potentially significant role within the bee habitat. Additional regional keys, such as the one provided here, may ease identification for the non-specialist and bring attention to the lesser-known species of both saprotrophs and pathogens.

**Table 1:** Distribution, host and substrate reports of *Ascosphaera* species in Europe.

*Ascosphaera* species	Distribution	Hosts	Substrate
*aggregata*	cosmopolitan	*Coelioxys echinata*	larvae [25]
		*Megachile pugnata*	larvae [38]
		*M. relativa*	larvae [39]
		*M. rotundata*	larvae [5], [23]
		*Osmia bicornis*	larvae [23]
*apis*	cosmopolitan	*Apis cerana*	larvae [40]
		*A. mellifera*	larvae [2], [26]
		*Xylocopa californica*	larvae [41]
*atra*	N. America, Europe, Oceania	*Chalicodoma aethiops*	pollen provisions [4]
		*M. rotundata*	pollen provisions [5], surface of chalkbrood larva [6], pollen inside the gut of a healthy larva [27]
		*Megachile* sp.	leaf-lining of brood cell*
		*—*	grass silage [6]
*callicarpa*	Denmark	*Chelostoma florisomne* *	larval feces*
*fimicola*	Denmark	*Cacoxenus indagator* (Diptera)*	larval feces*
		*O. bicornis*	cocoon*, pollen provisions*, larval feces [23]
*major*	Europe	*Anthophora pacifica*	larval feces [34]
		*Apis mellifera*	larvae [31], [33]
		*M. centuncularis*	larval feces [7], leaf-lining of brood cell [7], cocoon [7]
		*M. inermis*	cell lining [34]
		*O. bicornis* *	pollen provisions*
*proliperda*	Denmark, N. America	*M. centuncularis*	larvae [9]
	N. America	*M. rotundata*	larvae [35]
		*O. bicornis* *	larvae*
*tenax*	Denmark	*M. rotundata*	cocoon [24], larval feces [24], pollen provisions [24]
		*M. willughbiella*	cocoon [24], larval feces [24], pollen provisions [24]

* newly reported in present study.
